# Gut-associated metabolites and diabetes pathology: a systematic review

**DOI:** 10.3389/fendo.2025.1559638

**Published:** 2025-05-21

**Authors:** Katelyn L. Gough, Samantha J. Dando, Stephanie L. Teasdale, Beatrix Feigl, Flavia Huygens, Elise S. Pelzer

**Affiliations:** ^1^ School of Biomedical Sciences, Faculty of Health, Queensland University of Technology, Brisbane, QLD, Australia; ^2^ Centre for Immunology and Infection Control, Queensland University of Technology, Brisbane, QLD, Australia; ^3^ Centre for Vision and Eye Research, Queensland University of Technology, Brisbane, QLD, Australia; ^4^ Queensland Diabetes and Endocrine Centre, Mater Hospital Brisbane, Brisbane, QLD, Australia; ^5^ Mater Research Institute – University of Queensland, Faculty of Medicine, University of Queensland, Brisbane, QLD, Australia; ^6^ School of Clinical Medicine, Faculty of Health, Queensland University of Technology, Brisbane, QLD, Australia; ^7^ Queensland Eye Institute, Brisbane, QLD, Australia

**Keywords:** diabetes mellitus, metabolomics, systematic review, intestines, microbial metabolite

## Abstract

**Background:**

As the global prevalence of diabetes mellitus reaches epidemic proportions, research into new therapeutic targets that address the underlying pathomechanisms of the disease is essential. Recent studies have elucidated the fundamental role of intestinal metabolic pathways in human health and disease processes and yet, the underlying cause of metabolic dysregulation in diabetes is largely unknown. Therefore, this systematic review aimed to identify the intestinal metabolomic profiles associated with gestational diabetes mellitus, type 1 diabetes mellitus, pre-diabetes mellitus, and type 2 diabetes mellitus.

**Methods:**

A systematic review of databases and grey literature repositories identified primary literature published between 2005 and 2022, that investigated patterns of human- and microbial-derived metabolite concentration in individuals with diabetes.

**Results:**

Data extracted from thirty-four eligible studies revealed 272 metabolites that were associated with diabetes diseases; the majority correlated with incidence of type 2 diabetes mellitus only. Inter-study discrepancies were reported based on the biospecimen type used in metabolomic analyses, namely blood, stool, or urine.

**Conclusion:**

The results of this review emphasise the paucity of research investigating gestational and type 1 diabetes mellitus intestinal metabolic perturbations. Furthermore, the potential for inter-study bias in downstream metabolomic analyses based on sample type warrants further investigation.

## Introduction

The metabolites produced by enterocytes and commensal gut microbes directly regulate host immune responses and have the potential to contribute to metabolic inflammation (1). In healthy individuals, the metabolites contributed by eubiotic gut microbiota confer a plethora of benefits to the host, including nutrient synthesis and metabolism, inhibition of pathogen colonisation, regulation of host gene expression and metabolism, and regulation of the gut-immune axis (2–5). In contrast, the metabolites contributed by the dysbiotic gut microbiota promote pathogen colonisation and virulence, and dysregulate host physiology through modulation of intestinal mechanics and disturbed immune homeostasis (6). Through a multifaceted process of immunological crosstalk, the microbial metabolites released by dysbiotic microbiota act as immune-modulators that alter intestinal immune function and regulate the niches of microbe colonisation in the intestine; thereby creating conditions that favour the stabilisation of the dysbiotic configuration and inflammatory milieu (7, 8). It is therefore not surprising that an increasing volume of literature has implicated the metabolome of the gut microbiota in the development of diabetes and a range of metabolic disorders (9, 10).

However, discrepancies exist with respect to identifying the core diabetes-associated metabolites, and whether these metabolites help or hinder diabetic aetiology (11–13). Additionally, much of the current metabolomics research targets processes specific to type 2 diabetes mellitus (T2DM) only, given that T2DM accounts for approximately 90% of all diabetes diagnoses (4, 14–16). Therefore, research that also profiles the microbial and metabolomic profiles in individuals with gestational diabetes mellitus (GDM), and type 1 diabetes mellitus (T1DM) conditions is urgently needed to improve our understanding of diabetes pathophysiology.

To date, no systematic review has synthesised published intestinal metabolomic signature data encompassing all types of DM. In addressing this, we conducted this systematic review to collate, and recapitulate the main findings of studies that have investigated intestinal metabolite profiles in the context of GDM, T1DM, prediabetes (PreDM), and T2DM conditions. Furthermore, this review analysed the effect of study methods on metabolomic findings, by reviewing study findings in light of the collection techniques, and downstream analysis tools employed by researchers. This review ultimately aimed to synthesise and critically review existing literature so future diabetes research can be directed to the identification of pathophysiologically important metabolite markers.

## Methods

### Systematic review protocol

This systematic review was conducted in accordance with the core methods and processes outlined by the Preferred Reporting Items for Systematic Review and Meta-Analyses (PRISMA) 2020 Statement and the Cochrane Collaboration *Handbook for Systematic Reviews of Interventions* (17, 18).

### Eligibility criteria

This review included primary research that profiled the concentration of human- and microbial-derived metabolites in human cohorts with DM. Articles were not excluded based on the type of diabetes disease reported (PreDM, T1DM, T2DM, or GDM); provided the study only characterised the gut in a human population. Studies that characterised the gut metabolome of animals were excluded, based on the established anatomical, metabolic, and physiological differences between animal and human models (19–21).

Articles that were published over a seventeen-year period were screened (January 1, 2005, to June 30, 2022). This search period ensured research published prior to, and over the course of, the National Institutes of Health Human Microbiome Project (2007-2016) was screened for inclusion (22). Additionally, this commencement date correlates with the establishment of the HMDB and METLIN databases; the first standardised repositories of metabolomic data (23, 24). To ensure this review investigated the gut metabolome as the independent variable in the context of diabetes disease, only studies that reported on gut-associated metabolites were eligible for inclusion. That is, (i) metabolites that are utilised or produced as either intermediate or end-products of metabolic processes by human enterocytes or intestinal microbiota, (ii) reported in HMDB as originating from an intestinal biological location (whether directly from the intestinal organ, or from non-excretory fluid of faeces), or (iii) cited in existing literature as having a direct link with the gastrointestinal system. Only primary research literature was included for review; therefore, meta-analyses, review articles, systematic reviews, and commentaries were excluded, in addition to those that implemented an intervention (dietary, surgical, lifestyle) as part of the experimental methods. Similarly, only articles that were published (or translated for publication) in English were considered for inclusion in this systematic review.

### Information sources and database searches

Five electronic white literature databases were accessed for article screening; namely PubMed, Scopus, Excerpta Medica database (EMBASE), Web of Science, and the Cumulative Index to Nursing and Allied Health Literature (CINAHL). To ensure a comprehensive review of all existing primary literature was conducted, four grey literature databases were also consulted, spanning both National and International literature repositories; namely ProQuest Dissertations and Theses Global, Analysis and Policy Observatory (APO), Clinical trials.gov, and the International Network of Agencies for Health Technology Assessment Database (INAHTA).

**Figure 1:**
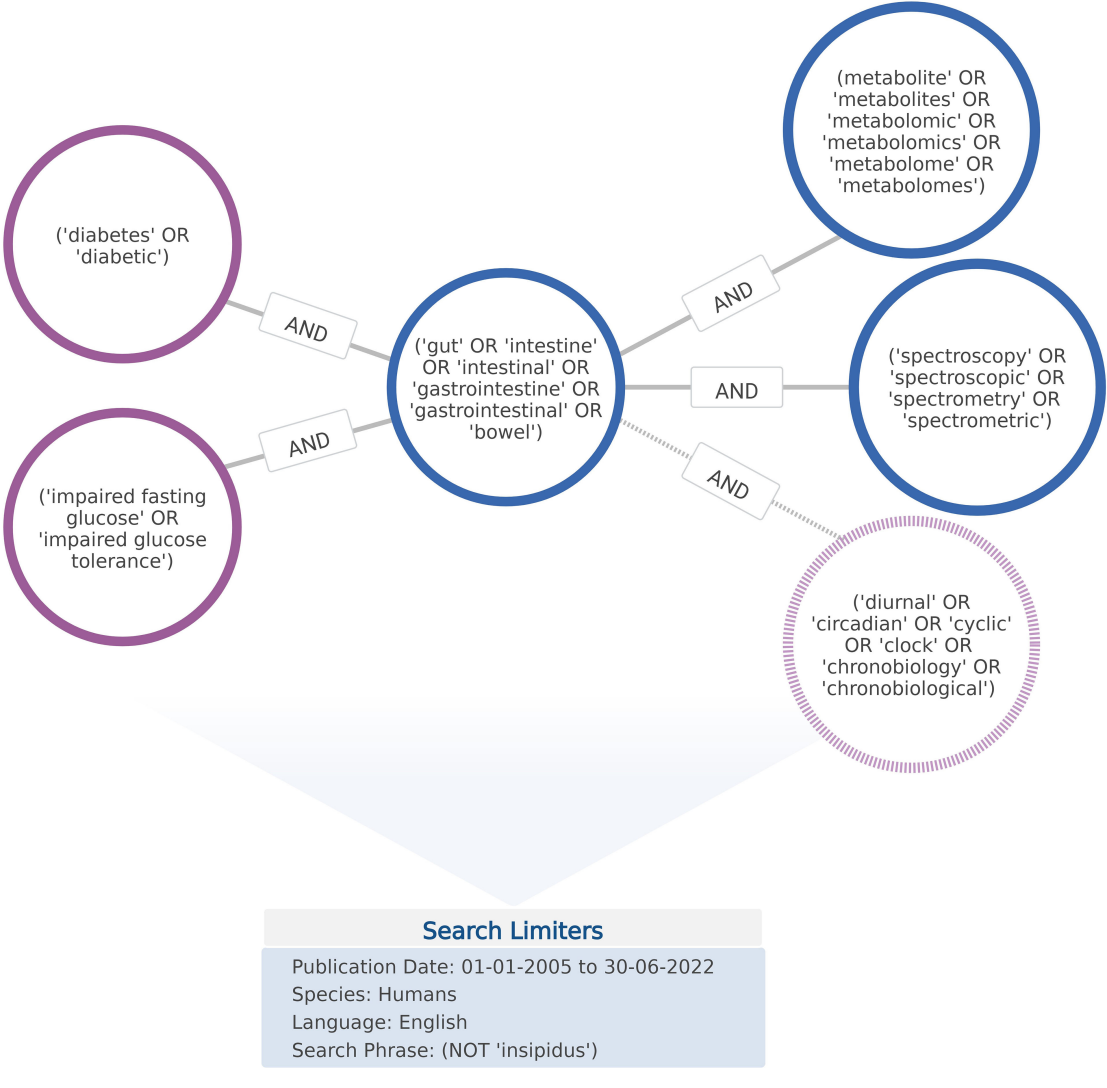
Terms and operators comprising search string queries applied to database and grey literature repository searches. Figure developed using BioRender software.

Databases were searched using key search terms included in the National Library of Medicine’s (NLM) Medical Subject Headings (MeSH) Index. In doing so, searches exploited current indexing in databases such as PubMed, ClinicalTrials.gov, and Embase that are modelled on, or indexed according to, MeSH classifications. As depicted in **Figure 1**, search strings were compiled from combinations of five main groups of search terms; (‘diabetes’ or ‘diabetic’); (‘impaired fasting glucose’ or ‘impaired glucose tolerance’); (‘gut’ or ‘intestine’ or ‘intestinal’ or ‘gastrointestine’ or ‘gastrointestinal’ or ‘bowel’); (metabolite’ or ‘metabolites’ or ‘metabolomic’ or ‘metabolomics’ or ‘metabolome’ or ‘metabolomes’); (‘spectroscopy’ or ‘spectroscopic’ or ‘spectrometry’ or ‘spectrometric’).

Search strategies were applied consistently across all five databases and all four grey literature repositories, with database-specific filters, limiters and expanders applied, as required. To ensure method repeatability, a comprehensive record of the inputs and outputs of every literature search was assembled in Microsoft Excel (v. 2019). Results from every targeted search were labelled and indexed into Smart Groups in referencing software, Endnote X9. Using both the ‘Find Duplicates’ function and manual screening, all duplicate records were then identified and excluded from the library, as per the PRISMA Statement process (17).

### Data collection and synthesis of results

At the completion of screening, one reviewer (KG) extracted specific data from the included articles and compiled these items into a Microsoft Excel (v. 2019) spreadsheet. The data items extracted from each article were: study and cohort descriptors (bibliographical details, diabetes disease investigated, experimental study design, cohorts and respective sizes, any inclusion/exclusion criteria applied to the study cohorts, and type of biological samples used for metabolome analysis), and analysis descriptors (overall metabolomics analysis approach, and metabolomics technique/s employed).

In addition to these data, the key metabolome data from each article was extracted and tabulated according to the metabolites that every publication reported as being significantly associated with a diabetes disease. Publication findings were synthesised according to whether metabolites were reported as being increased or decreased in cohorts with diabetes; in addition to the number of times each metabolite appeared in individual publications. This summation of metabolite count data highlights which targets warrant further investigation as potential diabetes biomarkers.

All metabolites identified by articles in this systematic review were annotated with the relevant HMDB primary accession number (25); to avoid simultaneous use of synonymous metabolites. Where a reported metabolite could not be matched to a HMDB entry, it was instead annotated with the compound identifier (CID) assigned by the NLM PubChem database. Furthermore, if the reported target did not match a specific HMDB entry (i.e., the target corresponds to a mixture of isomers or chemical species), then the HMDB ID of the most common compound variant was provided as an example.

To ensure consistency in reporting, metabolite targets from different publications were consolidated under a single compound name if listed as synonyms within the HMDB. Additionally, in keeping with the focus of this systematic review, only data pertaining to gut-derived metabolites were extracted from publications; that is, those metabolites known to originate from an intestinal biological location (whether directly from the intestinal organ, the gut microbiota, or from non-excretory fluid of faeces) or cited as having a direct link with the gastrointestinal system. Moreover, only metabolites identified using metabolomics tools are reported (i.e., not with commercially available laboratory assays including, for example, ELISAs). This ensured standardisation in the findings between different methodologies.

### Risk of bias in individual studies

Despite targeting a similar panel of gut-derived metabolites, significant methodological differences exist between research articles that investigate a facet of the intestinal metagenome. These differences extend from biological specimen collection techniques, to the methods and instruments used for downstream analyses. In addressing these discrepancies, this article aimed to evaluate if the employed methods of the included articles significantly impacted the metabolomic outputs reported. To achieve this, metabolomic findings were reported according to the type of biological specimen used in metabolomic analyses, and also based on the diabetes disease against which the findings were reported. Within these groups, Chi-Square tests of association were conducted to determine if the levels of any key gut-associated metabolites were significantly increased or decreased relative to (i) any diabetes disease type (i.e., T1DM, GDM, PreDM, or T2DM), or (ii) the reported sample collection method (i.e., blood, stool, or urine). These Chi-Square tests were conducted using R software, with *Post-hoc* pairwise tests conducted using the *chisq.posthoc.test* package to confirm significance of correlations between individual variables.

## Results

**Figure 2:**
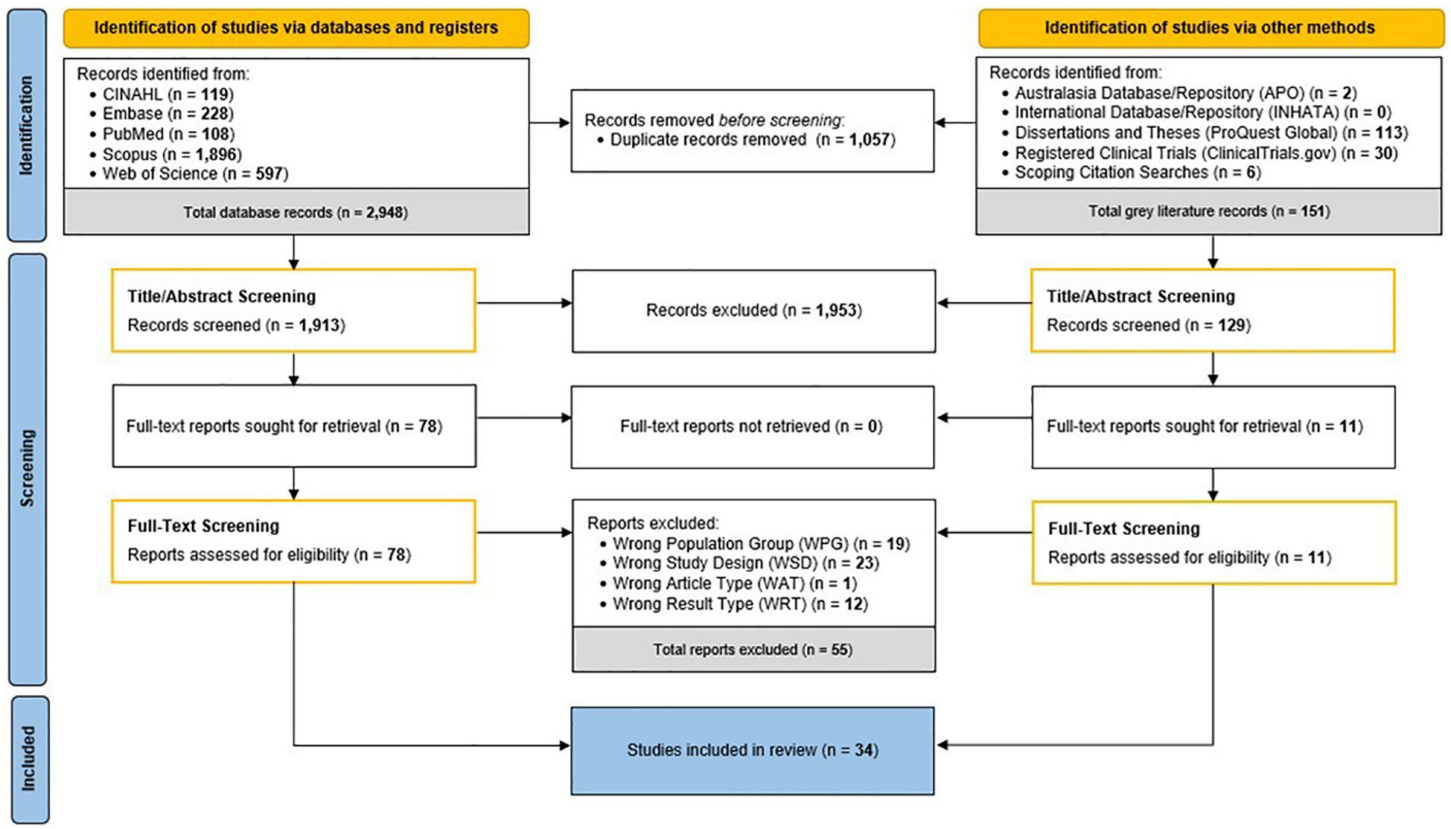
Workflow of systematic review identification, screening, and inclusion processes. APO, Analysis Policy Observatory; INHATA, International Network of Agencies for Health Technology Assessment; WAT, wrong article type; WPG, wrong population group; WRT, wrong result type; WSD, wrong study design. Schematic adapted from PRISMA 2020 Flow Diagram (17).

The search strategy originally yielded 3,099 records. After removal of duplicate records, 2,042 records were screened by title and abstract and 1,953 studies were excluded having not met the defined inclusion criteria. The remaining eighty-nine full text articles were screened; from which, a total of thirty-four articles were included as part of the systematic review. In summarising this, **Figure 2** depicts the study workflow for this review and details the number of articles included and excluded at each stage of the identification and screening processes.

The key cohort and analysis descriptors from the thirty-four reviewed records are summarised in **Table 1** according to the type of diabetes disease targeted; detailing the location, experimental design, sample type and associated storage conditions, study cohorts, and metabolomics methods incorporated.

**Table 1:** Key cohort and analysis descriptors of studies included in systematic review.

Study details	Sample	Cohort details and participant criteria		Metabolomic approach
T1DM studies				
(29) Spain Observational case-control study	Plasma^a^ Urine^a(24-hr)^	Age: 6–11 years. Sex: Mixed (27 female, 22 male). n = 49 (34 T1DM, 15 ND controls) Inclusion criteria: Under metabolic control Exclusion criteria: Psychological or DKA-associated diseases		Untargeted fingerprint analysis (i) LC-MS; HPLC and QTOF with FS, ESI+/- (ii) CE-MS; CE and TOF-MS with FS, ESI+
(28) Netherlands Observational case-control study	Plasma^a,b^ Stool^a,b^	Age: 18–65 years. Sex: Mixed (49 female, 54 male). n = 103 (53 T1DM, 50 ND controls) Inclusion criteria: Western European descent/dietary pattern, normal BMI^e^ Exclusion criteria: Altered microbiota composition, poor glucose regulation, microvascular complications		Targeted plasma SCFA analysis; LC-MS using HPLC and ion trap MS with SIM, ESI-. Targeted stool SCFA analysis; HPLC-UV
(27) Finland and Estonia Prospective longitudinal cohort study	Serum Stool	Age: From 0–36 months (longitudinal). Sex: Mixed (18 female, 15 male). n = 33 (4 T1DM, 7 T1DM-seroconverts^c^, 22 controls) Inclusion criteria: Positive HLA DR-DQ allele cord blood testing Prospective monitoring criteria: Infections, antibiotic use		Untargeted serum profiling; (i) UPLC-MS (lipids), (ii) GC×GC-TOFMS (metabolites). Targeted stool metabolite analysis; (i) U-HPLC with orbitrap MS, ESI+, FS, (ii) Hybrid quadrupole orbitrap MS with ESI+/-, FS
(26) Denmark Observational cross-sectional study	Plasma^a^	Age: ~46–72 years. Sex: Mixed (89 female, 122 male). n = 211 (161 T1DM, 50 ND controls) Inclusion criteria: >18 years. T1DM cohort; T1DM diagnosis (WHO criteria) and albuminuria status stratification Exclusion criteria: DKD, renal failure/dialysis/transplant, RAAS-blocking treatment change (≤1mo), antibiotic or immunosuppressive use		Targeted analyses; UHPLC MS/MS with ESI+/- and SR
GDM studies				
(36) China Observational case-control study	Plasma^a^	Age: No range defined. Sex: Female only (GDM study), n = 40 (20 GDM, 20 ND pregnant controls) Inclusion criteria: Diagnosis at 24–26 weeks (IADPSG criteria) Exclusion criteria: Pre-existing DM, CVD, infection, abnormal liver/kidney function		Untargeted profiling; ^1^H-NMR spectroscopy
(35) China Observational case-control study	Serum^a^	Age: ~26–35 years. Sex: Female only (GDM study). n = 52 (24 GDM, 28 ND pregnant controls) Inclusion criteria: Diagnosis at 24–28 weeks (IADPSG criteria), single birth. Exclusion criteria: Pregnancy complications, pre-existing DM, CVD, cerebrovascular disease, hypertension, GIT symptoms, anti-/pro-/prebiotic use		Targeted SCFA analysis; GC-MS with ESI+. Targeted BA, TMAO and TMAO-derivative analyses; UPLC-MS with ESI- and MRM
(34) China Nested case-control study	Serum^a,b^	Age: ~27–35 years. Sex: Female only (GDM study). n = 269 (131 GDM, 138 ND pregnant controls) Inclusion criteria: GDM diagnosis at 24–28 weeks (IADPSG criteria) Exclusion criteria: Alcohol consumption, pre-existing DM, infection, CVDs, abnormal liver/kidney function		Targeted FFA analysis; UPLC/QTOFMS with ESI+ (FFAs) and UPLC/TQMS with ESI- (BAs). Untargeted profiling; GC/TOFMS with electron impact ionisation and FS
(33) Czech Republic Observational case-control study	Plasma^d^	Age: 21–45 years. Sex: Female only (GDM study). n = 124 (84 GDM, 20 ND non-pregnant controls^c^, 20 ND pregnant controls) Selection criteria: Cohorts stratified based on trimester and FPG or PPG increase		Targeted SCFA analysis; LC-MS/MS using triple-quadrupole MS with ESI+ and MRM
(32) China Observational case-control study	Stool^a,b^	Age: No range defined. Sex: Female only (GDM study). n = 62 (31 GDM, 31 ND pregnant controls) Inclusion criteria: GDM diagnosis at 24–28 weeks (IADPSG criteria) Exclusion criteria: Pre-existing IFG, infection, abnormal liver/kidney function, CVD		Untargeted profiling; ^1^H-NMR spectral profiling
(31) Portugal Observational case-control study	Plasma^a^	Age: 18–44 years. Sex: Female only (GDM study). n = 93 (12 GDM, 32 preGDM^c^, 49 pregnant ND controls) Inclusion criteria: GDM (IASPSG criteria), clinical treatment not yet commenced		Untargeted metabolomic/lipidomic analysis; 1D ^1^H NMR spectroscopy
(30) China Observational case-control study	Stool^a^ Urine^a^	Age: ~26–35 years. Sex: Female only (GDM study). n = 107 (59 GDM, 48 pregnant ND controls) Inclusion criteria: GDM diagnosis at 24–28 weeks (WHO criteria) Exclusion criteria: Pre-existing DM, pregnancy complications, GIT symptoms, fetal chromosomal/structural abnormalities, anti-/pro-/prebiotic use within 1 month		Untargeted analysis; GC-MS
PreDM and T2DM studies				
(39) Germany Observational case-control study	Plasma^b^ Urine^a,b^	Age: ~35–59 years. Sex: Baseline characteristic not described. n = 51 (12 PreDM, 39 controls) Inclusion criteria: IGT diagnosis (WHO criteria) Exclusion criteria: CVD, kidney or liver diseases, or severe inflammation	Untargeted analysis; UPLC-QTOF-MS using reverse-phase UPLC coupled with QTOF-MS in ESI+/-	
(37) United States Observational cohort study	Plasma^a,b^	Age: 25–75 years (4-year longitudinal study). Sex: Mixed (55 female, 51 male). n = 106 Exclusion criteria: Pre-existing diseases (including renal, CVD or inflammatory disease), bariatric surgeries/liposuction Prospective monitoring criteria: Diabetes incidence, infection, and antibiotic use	Untargeted analysis; LC–MS/MS with HILIC and RPLC separation, ionisation (+/-) and FS	
(42) India Observational case-control study	Plasma^b^ Serum^b^	Age: 30–60 years. Sex: Mixed (42 female, 60 male). n = 102 (17 PreDM, 50 T2DM, 35 ND controls) Inclusion criteria: Cohorts stratified by HbA1c (ADA guidelines) and DM treatment Exclusion criteria: Antibiotic use, major GIT surgery, chronic clinical disorder	Targeted analysis; HPLC coupled with PDA detector	
(2) International repository Retrospective case-control study	Plasma^b^ Urine^b^	Age: ~36–73 years. Sex: Mixed (replication cohort: 321 female, 399 male; case cohort: female only). n = 3113 (115 T2DM, 822 PreDM, 2176 ND controls) Analysis criteria: Retrospective analysis of TwinsUK and KORA studies. Cohorts stratified based on fasting glucose. PreDM cohort; IFG diagnosis	Untargeted profiling; (i) UPLC-MS/MS with linear ion-trap and ESI+/-, (ii) GC-MS with electron impact ionisation	
(43) France, Germany and Denmark Retrospective case-control study	Serum^b^	Age: 39–67 years. Sex: Mixed (~973 female, 985 male). n = 1958 (765 T2DM, 654 PreDM, 539 ND controls) Inclusion criteria: Recruited from MetaCardis study. DM diagnosis (ADA criteria) Exclusion criteria: Non-metabolic CVD, antibiotic use, or abdominal surgery/radiotherapy/cancer	Targeted ImP analysis; UPLC-MS/MS	
(41) China Observational case-control study	Stool^a^(midstream)	Age: 30–60 years. Sex: Mixed (25 female, 35 male). n = 60 (20 T2DM, 20 PreDM, 20 ND) Inclusion criteria: Urumqi Uyghur subjects. DM diagnosis (AD guidelines). Exclusion criteria: Antidiabetic/lipid-lowering drugs, anti-/pre-/sym-/probiotic use, CVD, kidney disease, cancer, pregnancy/lactation, cognitive impairments, specialised diet, pets	Untargeted analyses; UPLC-Q-TOF/MS using orbitrap MS with ESI+/-	
(40) Finland Observational sub-cohort study	Serum^a,b^	Age: ~57–67 years (7-year follow-up). Sex: Male only. n = 531 Prospective monitoring criteria: Recruited from population-based METSIM cohort. Disease incidence, drug treatment, and health status monitored at follow-up. Subjects with T1DM or who developed T2DM were excluded	Targeted serum analysis; NMR spectroscopy. Targeted choline pathway analysis; LC-MS/MS with MRM+	
(38) China Observational case-control study	Plasma^a,b^	Age: ~50–74 years. Sex: Mixed (174 female, 119 male). n = 293 (114 T2DM, 81 PreDM, 98 ND controls) Inclusion criteria: > 40 y/o. DM cohorts defined using WHO diagnostic criteria Exclusion criteria: CVD, renal or autoimmune disease, cancer, antibiotic use	Untargeted lipidome analysis; LC-MS/MS using UPLC with QTOF and ESI+/-	
T2DM studies				
(58) Canada Observational case-control study	Plasma^a,b^	Age: ~23–63 years. Sex: Male only. n = 221 (67 T2DM, 106 IR ND controls^c^, 48 IS ND controls) Inclusion criteria: Stable metformin dose (≥3mo) Exclusion criteria: Smoking, high alcohol intake, other antidiabetic drug use (≥ 6wks), lipid-lowering medication withdrawal, other chronic diseases/infections, or altered endocrine/hepatic function	Targeted AA analysis; LC-MS using UHPLC with tandem quadrupole MS	
(57) China Observational case-control study	Plasma^a,b^	Age: 40–85 years. Sex: Mixed (77 female, 85 male). n = 162 (122 T2DM, 40 ND controls) Inclusion criteria: Han ethnicity. T2DM cohort; stratified by retinopathy status Exclusion criteria: Other diseases/infections, pregnancy/lactation, cognitive impairments, vegetarian diet, or anti-/probiotic use	Targeted TMAO quantitative analysis; LC-MS/MS with ESI+, MRM	
(56) International repository analysis Retrospective cohort study	Serum^b^	Age: ~49–73 years. Sex: Mixed (1,845 female, 695 male). n = 2,540 (case cohort: n=1,018 TwinsUK; replication cohort: n=1,522 ARIC) Analysis criteria: Recruited from TwinsUK and ARIC cohorts.	Untargeted profiling; (RP)UPLC-MS/MS with ESI+/- and HILIC/UPLC–MS/MS with ESI-	
(55) Denmark Observational case-control study	Serum^a,b^	Age: ~50–66 years. Sex: Mixed (187 female, 179 male). n = 366 (75 T2DM, 277 ND controls) Inclusion criteria: Caucasian Danish subjects recruited from MetaHIT study Exclusion criteria: GIT disease, bariatric surgery, immune-altering medications, antibiotic use	Combined targeted-untargeted analysis; GC × GC-TOFMS (metabolome) and UHPLC-QTOFMS with ESI+ (lipidome)	
(54) Netherlands Observational case-control study	Plasma Urine	Age: ~41–69 years. Sex: Mixed (~64 female, 74 male). n = 138 (52 T2DM, 34 IBD^c^, 52 ND controls) Inclusion criteria: Recruited from CODAM study. Caucasian ethnicity, >40 years, one or more T2DM risk factor	Targeted lactate analysis; UPLC-MS/MS using RPLC with MRM-	
(53) United States Prospective longitudinal cohort study	Plasma^b^	Age: 25–75 years (<8-year follow-up). Sex: Mixed (55 female, 54 male). n = 109 Inclusion criteria: High BMI^e^, normal/PreDM OGTT Exclusion criteria: Eating disorder or psychiatric disease, hypertriglyceridemia, uncontrolled hypertension, high alcohol intake, pregnancy/lactation, bariatric surgery	Untargeted metabolome analysis; LC-MS using RPLC/HILIC with ESI and FS. Untargeted lipidome analysis; DMS with quadrupole ion-trap (Lipidyzer platform).	
(52) China Observational case-control study	Plasma^b^	Age: ~43–65 years. Sex: Mixed (862 female, 1,832 male). n = 2694 (1346 T2DM, 1348 ND controls) Inclusion criteria: Han ethnicity, newly diagnosed T2DM (WHO criteria) Exclusion criteria: <30 y/o, ≥ 40 kg/m^2^, hyperlipidaemia treatment, systemic/acute illness, or chronic inflammatory/infective disease	Targeted TMAO analysis; LC-MS/MS using stable isotope dilution LC with online MS/MS	
(51) Germany Observational case-control study	Plasma^a,b^ Serum^a,b^	Age: 55–79 years. Sex: Male only. n = 100 (40 T2DM, 60 ND controls) Inclusion criteria: >54 years, recruited from the population-based KORA F3 cohort	Combined targeted and untargeted analysis; Serum - UHPLC/MS/MS^2^ (Metabolon) and ESI-MS/MS (Biocrates). Plasma - ^1^H-NMR spectroscopy (Chenomx).	
(50) United States Observational prospective cohort study	Plasma^a,b^	Age: ~37–75 years (5-year follow-up). Sex: Mixed (~712 female, 804 male). n = 1516 (1216 T2DM, 300 ND controls) Inclusion criteria: Prospectively recruited based on T2DM status-cardiac event risk. Adverse cardiac event/all-cause mortality investigated at follow-up. Exclusion criteria: Acute coronary syndrome (<30 days)	Targeted TMAO analysis; LC-MS/MS; stable isotope dilution LC with online triple quadrupole MS	
(49) Indonesia Observational cross-sectional study	Stool^a^ (methanol treatment)	Age: 34–63 years. Sex: Male only. n = 75 (25 T2DM, 21 obese^c^, 29 ND) Inclusion criteria: Adult residents of Yogyakarta city (>3yrs) Exclusion criteria: Local or systemic disease or infection. Defined medication, antimicrobials, intensive disease therapy, or high pro-/prebiotic use	Targeted SCFA analysis; ^1^H-NMR spectroscopy. Targeted BA analysis; LC-MSMS with ESI-, MRM and SIM	
(48) Finland Observational sub-cohort study	Plasma^b^	Age: 50–64 years (7.4-year follow-up). Sex: Male only. n = 5169 Prospective monitoring criteria: Disease incidence, drug treatment, and health status monitored. Recruited from METSIM cohort; baseline T2DM cases excluded	Untargeted profiling; UHPLC-MS/MS with heated ESI	
(46) China Observational case-control study	Stool^b,d^	Age: 40–58 years. Sex: Mixed (47 female, 53 male). n = 100 (65 T2DM, 35 ND controls) Exclusion criteria: Constipation/diarrhoea, hepatitis, high alcohol intake, smoking, pro-/antibiotic use. T2DM cohort; metformin or TCM treatment	Targeted SCFA analysis; GC-MS. Untargeted endogenous metabolite analysis; UPLC-QTOF-MS with ESI +/-	
(47) China Observational case-control study	Serum^a,b^ Stool^a,b^		Targeted SCFA analysis; Triple quadrupole GC-MS with electron impact, SIM. Targeted BA analysis; UPLC-MS/MS with MRM, ESI-	
(45) China Observational cross-sectional study	Serum^a,b^ Stool (crushed)	Age: ~43–69 years. Sex: Mixed (32 female, 58 male). n = 90 (30 T2DM/DKD^c^, 30 T2DM, 30 ND controls) Inclusion criteria: T2DM (ADA criteria), DKD Exclusion criteria: >75 years, special dietary habit, laxative or anti-/probiotic use, yogurt consumption within 2 months, obesity, infection, renal/liver/gut bacteria-altering diseases	Targeted SCFA analysis; GC-MS with SIM	
(44) China Observational case-control study	Stool^a^	Age: ~51–68 years. Sex: Mixed (21 female, 29 male). n = 50 (21 T2DM/DR^c^, 14 T2DM, 15 ND controls) Inclusion criteria: T2DM (ADA criteria), DR (diagnosed by SLO/FA), normal diet/bowel movements, stable metformin dose Exclusion criteria: Other eye diseases, corticosteroid/pro-/antibiotic use, GIT surgery, autoimmune disease, hypertension, obesity, tumours, organ transplant	Untargeted analyses; UHPLC-MS; UHPLC coupled with orbitrap MS	

Studies presented alphabetically according to DM disease type. ^a^Samples stored between -86 and -80°C prior to analyses; ^b^Fasting sample; ^c^Results of these cohorts not included in comparative analyses of DM cohorts relative to ND control cohorts; ^d^Samples stored at -20°C short-term; ^e^Normal BMI defined as 18.5–25 kg/m^2^. ADA, American Diabetes Association; BA, bile acid; BMI, body mass index; CE-MS, capillary electrophoresis-mass spectrometry; CVD, cardiovascular disease; DKD, diabetic ketoacidosis; DR, diabetic retinopathy; ESI, electrospray ionisation; FA, fluorescein angiography; FFA, free fatty acid; FPG, fasting plasma glucose; GDM, gestational diabetes mellitus; GIT, gastrointestinal tract; HILIC, hydrophilic interaction; HPLC, high-performance liquid chromatography; IADPSG, International Association of the Diabetes and Pregnancy Study Groups; IFG, impaired fasting glucose; LC-MS, liquid chromatography-mass spectrometry; METSIM, METabolic Syndrome in Men; MRM, multiple reaction monitoring; MS, mass spectrometry; ND, non-diabetes; NMR, nuclear magnetic resonance; PDA, photo diode array; PPG, postprandial glucose; PreDM, prediabetes mellitus; QTOF, quadrupole time of flight; RAAS, renin-angiotensin-aldosterone system; RPLC, reverse-phase liquid chromatography; SCFA, short-chain fatty acid; SIM, selected ion monitoring; SLO, scanning laser ophthalmoscopy; SRM, selected reaction monitoring; T1DM, type 1 diabetes mellitus; T2DM, type 2 diabetes mellitus; TMAO, trimethylamine N-oxide; UPLC, ultra performance liquid chromatography; -UV, ultraviolet (spectroscopy); WHO, World Health Organisation.

**Table 2** lists the gut-associated metabolites reported as being significantly associated with diabetes. In this table, metabolites are reported alongside the sample type/s used in sourcing analysis, and colour-coded depending on whether the target was found to be increased or decreased in DM cohorts relative to non-diabetes (ND) control cohorts. Additionally, all 272 metabolites are listed with the corresponding HMDB primary accession number, to facilitate cross-referencing with the chemical repository.

**Table 2:** Gut-associated metabolites reported as significantly associated with diabetes diseases.

Metabolite	HMBD ID	Association with diabetes disease
Amino acids, peptides, and analogues		
Aceturic acid	HMDB0000532	↓ T2DM blood (2)
Acetyl-arginine	HMDB0004620	↑ T1DM urine (29)
Acetyl-aspartic acid	HMDB0000812	↑ GDM blood (34)
Acetyl-L-tyrosine	HMDB0000866	↑ T2DM stool (46)
Alanine	HMDB0001310	↓ T1DM blood (26), GDM blood (31) ↑ PreDM blood (37), GDM blood (34)^c^, T2DM blood (55, 58)
Allantoic acid	HMDB0001209	↑ GDM stool (30)
2-Aminobutanoic acid	HMDB0000650	↑ GDM blood (34)
γ-Aminobutyric acid	HMDB0000112	↑ GDM blood (34)
Aminomalonic acid	HMDB0001147	↑ GDM blood (34)
Arginine	HMDB0303361	↓ T2DM blood (58)
L*-*Aspartic acid	HMDB0000191	↑ T2DM blood (55), T2DM stool (44)
Betaine	HMDB0000043	↓ GDM blood (31) ↑ T2DM blood (50)
Cinnamoyl glycine	HMDB0011621	↓ PreDM blood (37), T2DM blood (56)
Citrulline	HMDB0000904	↓ T2DM blood (2, 58) ↑ T1DM blood (26)
Creatine	HMDB0000064	↑ T2DM blood (48, 53)^b^
Creatinine	HMDB0000562	↓ GDM blood (31) ↑ T2DM blood (51)
Cysteine	HMDB0000574	↓ GDM blood (34)
Cysteine-glycine	HMDB0000078	↓ GDM urine (30)
Cystine	HMDB0000192	↑ T1DM urine (29), T2DM blood (53)^b^
N-(4,5-Dihydro-1-methyl-4-oxo-1H-imidazol-2-yl) alanine	HMDB0034912	↓ T2DM stool (44)
Dimethylarginine	HMDB0003334 HMDB0001539	↓ T2DM blood (2) ↑ T1DM blood (26)
Dimethylglycine	HMDB0000092	↑ T2DM blood (48)
Ectoine	HMDB0240650	↓ T2DM blood (53)^b^
Glutamic acid	HMDB0000148^^^	↑ GDM blood (34), T2DM blood (55, 58)
Glutamine	HMDB0000641	↓ GDM urine (30), T2DM blood (58)
N-γ-L-Glutamyl-D-alanine	HMDB0036301	↑ T2DM stool (44)
γ-Glutamyl-glutamine	HMDB0029147	↑ PreDM stool (41), T2DM stool (41, 44)
γ-Glutamyl-histidine	HMDB0029151	↓ T2DM blood (53)^b^
γ-Glutamyl- isoleucine	HMDB0011170	↑ T2DM blood (51)
Glutamyl-lysine	HMDB0004207	↑ T2DM stool (44)
Glutamyl-valine	HMDB0028832	↑ T2DM blood (51)
Glycine	HMDB0000123	↓ GDM urine (30), T2DM blood (58)
Glycyl-histidine	HMDB0028843	↑ T2DM stool (51)
Histidine	HMDB0000177	↓ T2DM blood (42, 58)
Homocitrulline	HMDB0000679	↑ T1DM blood (26), T2DM blood (51)
Homocysteine	HMDB0000742	↑ GDM stool (32)
Leucine & Isoleucine^a^	HMDB0000687 HMDB0000172	↓ T1DM blood (26) ↑ T1DM blood (27), T1DM urine (29), GDM blood (34), PreDM blood (2, 40)^b^, T2DM blood (2, 42, 51, 55, 58)
Lysine	HMDB0000182	↑ T1DM urine (29), PreDM stool (41)
3-Methoxytyrosine	HMDB0001434	↑ GDM urine (30)
Methionine	HMDB0000696	↓ PreDM blood (42) ↑ T2DM blood (42)
N-Methyl-glutamic acid	HMDB0062660	↓ GDM urine (30)
N-Methyl-proline	HMDB0094696	↓ T2DM blood (53)^b^
Noropthalmic acid	HMDB0005766	↓ T2DM stool (44)
Phenylacetylglutamine	HMDB0006344	↓ PreDM urine (39)^d^ ↑ T2DM blood (51)
Phenylalanine	HMDB0000159	↓ T1DM blood (26) ↑ T2DM blood (51, 58)
Proline	HMDB0000162	↓ GDM blood (31) ↑ GDM blood (36), T2DM blood (2, 55)
Proline betaine	HMDB0004827	↓ T2DM blood (53)^b^, T2DM stool (44)
Trimethyllysine	HMDB0001325	↓ T2DM blood (53)^b^
Tyrosine	HMDB0000158	↓ T1DM blood (26) ↑ PreDM stool (41), T2DM blood (42, 58)
Valine	HMDB0000883	↑ T1DM blood (27), T1DM urine (29), GDM stool (30), GDM blood (34), PreDM blood (40)^b^, T2DM blood (2, 42, 55)
Azoles		
Imidazole propionate	HMDB0002271	↑ PreDM blood (43), T2DM blood (43, 56)
Benzoic acids and derivatives		
2-(Ethylamino)-4,5-dihydroxybenzamide	HMDB0032852	↓ T2DM stool (44)
Gentisic acid	HMDB0000152	↓ T2DM stool (44)
Hippuric acid	HMDB0000714	↓ PreDM blood (37), PreDM urine (39)^d^
(4-Hydroxybenzoyl)choline	HMDB0029559	↓ T2DM stool (44)
3-Hydroxyhippuric acid	HMDB0006116	↓ PreDM urine (39)^d^
Salicyluric acid	HMDB0000840	↑ T2DM blood (48)
Benzopyrans		
3'-Deaminofusarochromanone	HMDB0041328	↓ T2DM stool (44)
Bi- and oligothiophenes		
5-(1-Propynyl)-5'-vinyl-2,2'-bithiophene	HMDB0038430	↓ T2DM stool (44)
Carbohydrates and carbohydrate conjugates		
N-Acetylgalactosamine-4-sulphate	HMDB0000781	↓ GDM stool (32)
N-Acetyl-D-glucosamine	HMDB0000215	↑ T2DM blood (55)
N-Acetylhexosamine	HMDB0248228	↑ T2DM blood (51)
1,5-Anhydroglucitol	HMDB0002712	↓ T2DM blood (2, 51)
Arabinose	HMDB0000646	↑ T2DM blood (2)
Ethyl glucuronide	HMDB10325	↑ T2DM blood (53)^b^
Erythritol	HMDB02994	↑ PreDM blood (2), T2DM blood (53)^b^
Fructose	HMDB0000660	↑ PreDM blood (2), T2DM blood (2)
Fucitol	HMDB0304954	↓ PreDM blood (37)
Galactitol	HMDB0000107	↓ GDM urine (30) ↑ GDM blood (36)
D-Galactose	HMDB0000143	↑ GDM urine (30)
Glucose	HMDB0000122	↑ GDM urine (30), PreDM blood (2), T2DM blood (2, 51)
Glucuronic acid	HMDB0000127	↑ T2DM blood (51)
Glycerol	HMDB0000131	↓ GDM blood (36)
Maltose	HMDB0000163	↓ GDM blood (34) ↑ T2DM blood (51)
Mannose	HMDB0000169	↑ GDM blood (34), PreDM blood (2), T2DM blood (2, 51)
Threitol	HMDB04136	↑ GDM blood (34), T2DM blood (53)^b^
Threonic acid	HMDB0000943	↓ GDM blood (34)
Trehalose	HMDB0000975	↓ GDM urine (30)
Carboxylic acids and derivatives		
Acetic acid	HMDB0000042	↓ T1DM blood (28), T2DM stool (46) ↑ T2DM blood (47)
Isobutyric acid	HMDB0001873	↓ T2DM stool (46) ↑ GDM blood (35), T2DM blood (47)
Isocitric acid	HMDB0000193	↓ GDM stool (30)
Methylmalonic acid	HMDB0000202	↓ GDM blood (36)
Propionic acid	HMDB0000237	↓ T1DM blood (28), T2DM stool (45, 46) ↑ T2DM blood (47)
Succinic acid	HMDB0000254	↓ T2DM stool (44)
Coumarins and derivatives		
Protorifamycin I	HMDB0039057	↑ PreDM stool (41)
Dihydrofurans		
2-Methylascorbic acid	HMDB0240294	↓ GDM urine (30)
Dipeptides and hybrid peptides		
Carnosine	HMDB0000033	↓ T2DM stool (44)
Lysyl-Cysteine	18218224	↑ T1DM blood (29)
Valyl-asparaginyl-alanine	145458842	↑ T1DM blood (29)
Fatty acids and conjugates		
Adrenic acid	HMDB0002226	↑ T1DM blood (29), PreDM blood (30)
Arachidic acid	HMDB0002212	↑ GDM blood (34)
Arachidonic acid	HMDB0001043	↑ GDM blood (34), PreDM blood (30)
Butyrate	HMDB0000039	↓ T2DM stool (46) ↑ T2DM blood (47)
Capric acid	HMDB0000511	↑ T2DM blood (53)^b^
Caproic acid	HMDB0000535	↓ T2DM blood (47, 51), T2DM stool (46) ↑ GDM blood (35)
Citraconic acid	HMDB0000634	↓ GDM urine (30)
5,6-Dichlorotetradecanoic acid	101254179	↑ PreDM blood (38), T2DM blood (38)
10,20-Dihydroxyeicosanoic acid	HMDB0031923	↓ PreDM blood (37), T2DM blood (53)^b^
9,10-Dihydroxy-12-octadecenoic acid	HMDB0004704	↓ T2DM stool (46)
Docosapentaenoic acid	HMDB0006528	↑ GDM blood (34)
Docosatrienoic acid	HMDB02823	↑ GDM blood (34)
Dodecanoic acid	HMDB0000638	↑ GDM blood (34)
5-Dodecenoic acid	HMDB0000529	↓ T2DM blood (2) ↑ PreDM stool (41)
Eicosadienoic acid	HMDB05060	↑ GDM blood (34)
Eicosapentaenoic acid	HMDB0001999	↓ T2DM stool (44)
Eicosatrienoic acid	HMDB0002925	↑ GDM blood (34), T2DM blood (53)^b^
2-Ethylhexanoic acid	HMDB0031230	↑ GDM blood (34)
Gondoic acid	HMDB0002231	↑ GDM blood (34)
Heptadecanoic acid	HMDB0002259	↑ GDM blood (34)
Heptadecenoic acid	HMDB60038	↓ T2DM blood (2) ↑ GDM blood (34), T2DM blood (53)^b^
Heptanoic acid	HMDB0000666	↓ T2DM blood (2, 51)
Hexadecadienoic acid	HMDB0302694	↑ GDM blood (34), T2DM blood (53)^b^
Hexadecatrienoic acid	HMDB0302991	↑ T2DM blood (53)^b^
2-Hydroxyadipic acid	HMDB0000321	↑ PreDM stool (41)
2-Hydroxydecanoate	HMDB0094656	↓ T1DM blood (29), GDM urine (30)
Hydroxyisocaproic acid	HMDB0000746	↓ T2DM blood (53)^b^
Hydroxyisovaleric acid	HMDB0000407^^^	↓ T2DM blood (53)^b^
Hydroxypalmitoleic acid	46235676^^^	↑ T2DM blood (53)^b^
Isovaleric acid	HMDB0000718	↓ T2DM stool (46) ↑ GDM blood (35), T2DM blood (47)
Myristic acid	HMDB0000806	↓ T2DM blood (2) ↑ T2DM blood (53)^b^
Myristoleic acid	HMDB0002000	↓ T2DM blood (2) ↑ T2DM blood (53)^b^
Nonadecenoic acid	HMDB0302287	↑ GDM blood (34)
Octadecanedioic acid	HMDB0000782	↑ T2DM blood (53)^b^
9-Octadecenoic acid	HMDB62703	↑ GDM blood (34)
Oleic acid	HMDB0000207	↑ T1DM blood (29)
Palmitic acid	HMDB0000220	↑ T1DM blood (29), GDM blood (34), PreDM blood (39)
Palmitoleic acid	HMDB03229	↓ T2DM blood (2) ↑ T1DM blood (29), GDM blood (34), T2DM blood (53)^b^
Pelargonic acid	HMDB0000847	↓ T2DM blood (2, 51)
Pentadecanoic acid	HMDB0000826	↓ T2DM blood (2) ↑ T2DM blood (53)^b^
Stearic acid	HMDB0000827	↑ PreDM blood (39)
Tetradecanedioic acid	HMDB0000872	↓ PreDM blood (37), T2DM blood (53)^b^
9E-Tetradecenoic acid	HMDB0062248	↑ GDM blood (34)
Tridecanoic acid	HMDB0000910	↕ T2DM blood (53)^b^
Valeric acid	HMDB0000892	↓ T2DM blood (47), T2DM stool (46) ↑ GDM blood (35)
Fatty acid esters		
Acylcarnitine C10:3	Not Defined	↑ T2DM slool (53)^b^
Decanoylcarnitine	HMDB0000651	↑ PreDM urine (39)^d^
Dibutyl decanedioate	HMDB0041220	↓ GDM stool (32)
Glutarylcarnitine	HMDB0013130	↑ T2DM blood (53)^b^
Hexanoylcarnitine	HMDB0000756	↑ T2DM blood (53)^b^
Hydroxybutyrylcarnitine	HMDB0013127	↑ PreDM blood (38), T2DM blood (38)
Methyl hexadecenoic acid	HMDB0061859	↓ T2DM blood (2)
Octanoylcarnitine	HMDB0000791	↓ T2DM blood (2) ↑ PreDM urine (39)^d^
Palmitoylcarnitine	HMDB0000222	↑ T2DM stool (46)
Valerylcarnitine	HMDB0013128	↓ T2DM blood (53)^b^
Fatty acyls		
Deoxyglucose	HMDB0062477	↑ T2DM blood (51)
1-Octen-3-yl glucoside	HMDB0032959	↓ T2DM stool (44)
N-(3-oxooctanoyl)-homoserine lactone	127293	↓ T2DM blood (38)
Thromboxane B3	HMDB0005099	↓ T2DM stool (44)
Furanoisoflavonoids		
Cristacarpin	HMDB0034025	↑ T2DM stool (41)
Glycerolipids		
Diacylglycerol	HMDB0242173	↑ T2DM stool (46)
Glyceryl monooleate	HMDB0254854	↑ GDM blood (34)
1-Myristoylglycerol	HMDB0304951	↑ T2DM blood (48)
1-Oleoylglycerol	HMDB0011567	↑ T2DM blood (48)
1-Palmitoleoylglycerol	9883914	↑ T2DM blood (48)
Triglycerides	HMDB0005356^^^	↓ PreDM blood (38) ↑ T1DM blood (27) ↕ T2DM blood (38, 55)
Glycerophospholipids		
Glycerophosphocholine	HMDB0000086	↓ T2DM blood (51)
Linoleoyl lysophosphatidylcholine	HMDB0010386	↑ T2DM blood (48)
Lysophosphatidylcholines	HMDB0010379^^^	↓ T1DM blood (29), PreDM blood (39), T2DM blood (38, 53)^b^ ↑ PreDM stool (41), T2DM blood (55), T2DM stool (46)
Lysophosphatidylethanolamines	HMDB0011472^^^	↑ T2DM blood (53)^b^
Phosphatidylcholine	HMDB0000564^^^	↓ PreDM blood (38), T2DM blood (38, 55) ↑ T1DM blood (29), T2DM blood (51, 53)^b,c^, T2DM stool (46)
Phosphatidylethanolamine	HMDB0060501	↑ T1DM blood (29), T2DM blood (53)^b^, T2DM stool (41) ↕ T2DM blood (55)^e^
Phosphatidylinositol	HMDB0009783^^^	↑ T2DM blood (53)^b^
Phosphatidylserine	HMDB0014291	↑ PreDM blood (38), T2DM blood (38)
Hydroxy acids and derivatives		
2-Hydroxybutyric acid	HMDB0000008	↑ T1DM blood (26), GDM blood (34), PreDM blood (2), T2DM blood (48)
3-Hydroxybutyric acid	HMDB0000011	↑ T1DM blood (26), GDM blood (33), T2DM blood (51)
3-Hydroxycapric acid	HMDB0002203	↓ PreDM blood (37)
Lactic acid^a^	HMDB0001311 HMDB0000190	↑ T2DM blood (2, 54, 55), T2DM urine (54)
Malic acid	HMDB0000156	↓ GDM stool (32) ↑ GDM blood (34), T2DM blood (2)
Indoles and derivatives		
β-Carboline	HMDB0012897	↓ T2DM stool (44)
4-Formylindole	HMDB0341228	↓ PreDM blood (37)
Hydroxyindoleacetate	HMDB0000763	↓ GDM stool (30)
Indole-3-carboxylic acid	HMDB0003320	↓ T2DM stool (44)
Indolelactic acid	HMDB0000671	↓ PreDM blood (37)
Indole propionic acid	HMDB0002302	↓ T2DM blood (53)^b^, T2DM stool (44)
Tryptophan	HMDB0000929	↓ T1DM blood (26) ↑ PreDM urine (39)^d^, T2DM blood (42)
Keto acids and derivatives		
Ketoisocaproic acid	HMDB0000695	↑ GDM blood (34), PreDM blood (2), T2DM blood (2, 55)
Ketoisovaleric acid	HMDB0000019	↑ PreDM blood (2)
3-Methyl-2-oxovaleric acid	HMDB0000491	↑ PreDM blood (2), PreDM urine (2), T2DM blood (2), T2DM urine (2)
Octenoylcarnitine	HMDB0240723	↑ T2DM blood (38)^b^
Oxoglutaric acid	HMDB0000208	↑ T2DM blood (55)
2-Oxoisovaleric acid	HMDB0000019	↑ T2DM blood (55)
Pyruvic acid	HMDB00243	↑ GDM blood (34), T2DM blood (53, 55)^b^
Lactones		
Matricin	HMDB0036643	↑ PreDM stool (41)
Lineolic acids and derivatives		
Cucurbic acid	HMDB0029388	↑ PreDM stool (41)
Hydroxylinolenic acid	HMDB0011108	↑ T2DM blood (53)^b^
Linoleic acid	HMDB0000673	↑ T1DM blood (29), GDM blood (34)
Linolenic acid	HMDB0001388	↑ T1DM blood (29), GDM blood (34)
γ-Linolenic acid	HMDB0003073	↑ GDM blood (34)
Macrolides and analogues		
Epothilone A	HMDB0251873	↑ PreDM stool (41)
Organic sulfuric acids and derivatives		
Indoxyl sulfate	HMDB0000682	↑ T2DM blood (51)
Methyl-4-hydroxybenzoate sulfate	HMDB0168668	↓ PreDM blood (37)
Organonitrogen compounds		
Choline	HMDB0000097	↑ T2DM blood (50)
Phytosphingosine	HMDB0004610	↓ T2DM stool (44)
Trimethylamine-N-oxide	HMDB0000925	↓ GDM blood (31) ↑ T2DM blood (50, 52, 57)
Organooxygen compounds		
Butanone	HMDB0000474	↑ GDM stool (32)
3-Dehydroshikimate	HMDB0304122	↓ GDM urine (30)
Kynureinine	HMDB0000684	↑ T2DM blood (51)
Methanol	HMDB0001875	↓ GDM blood (31)
Phenols		
Capsaicin	HMDB0002227	↓ T2DM stool (44)
p-Hydroxyfelbamate	HMDB0060669	↓ T2DM stool (44)
Tyrosol	HMDB0004284	↓ T2DM stool (44)
Phenylpropanoic acids		
Hydrocinnamic acid	HMDB0000764	↓ T2DM blood (56) ↑ T2DM blood (55)
Hydroxyphenyllactic acid	HMDB0000755	↑ T2DM blood (48)
Prenol lipids		
Anhydrorhodovibrin	5368308	↑ PreDM stool (41)
ar-Artemisene	HMDB0039155	↓ T2DM stool (44)
Astaxanthin	HMDB0002204	↓ T2DM stool (44)
Dehydrovomifoliol	HMDB0036819	↑ PreDM stool (41)
Galbanic acid	HMDB0030163	↓ T1DM blood (29)
Ginkolide C	HMDB0036860	↓ T2DM stool (44)
Isobornyl propionate	HMDB0038249	↓ T2DM stool (44)
Manoalide	HMDB0254329	↑ PreDM stool (41)
Oleanolic acid	HMDB0002364	↓ T2DM stool (46)
Quassin	HMDB0036587	↑ PreDM stool (41)
Ubiquinone-2	HMDB0006709	↓ T2DM stool (44)
Purines and purine derivatives		
7-Methylguanine	HMDB0000897	↓ T2DM stool (44)
1-Methylhypoxanthine	HMDB0013141	↓ T2DM stool (44)
Methyluric acid	HMDB0003099^^^	↓ PreDM urine (39)^d^
Methylxanthine	HMDB0010738^^^	↓ PreDM urine (39)^d^
Uric acid	HMDB0000289	↓ PreDM urine (39)^d^ ↑ PreDM blood (2), T2DM blood (48)
Xanthine	HMDB0000292	↑ PreDM urine (39)^d^, T2DM blood (48), T2DM stool (44)
Purine nucleosides		
Adenosine	HMDB0000050	↓ T2DM stool (44)
N6-Carbamoyl-L-threonyladenosine	HMDB0041623	↑ T2DM blood (53)^b^
N2,N2-Dimethylguanosine	HMDB0004824	↑ T2DM blood (53)^b^
1-Methyladenosine	HMDB0003331	↑ T2DM blood (53)^b^
Pyridines and derivatives		
N-Methylnicotinamide	HMDB0003152	↑ T1DM blood (26)
Niacinimide	HMDB0001406	↓ T2DM stool (44)
Nicotinic acid	HMDB0001488	↓ GDM urine (30)
Pyrimidine nucleosides		
Cytidine	HMDB0000089	↓ T2DM stool (44)
Pyrimidines and derivatives		
Cytosine	HMDB0000630	↓ T2DM stool (44)
3-Methylcytosine	HMDB0011601	↓ T2DM stool (44)
Thymine	HMDB0000262	↑ T2DM stool (44)
Uracil	HMDB0000300	↓ T2DM stool (44)
Quinolines and derivatives		
Kynurenic acid	HMDB0000715	↓ GDM stool (30) ↑ T2DM blood (48)
Xanthurenic acid	HMDB0000881	↑ T2DM blood (48)
Sphingolipids		
Ceramides	HMDB0004949^^^	↕ T2DM blood (55)
Sphingomyelin	HMDB12096	↓ T1DM stool (27), T2DM blood (2, 51, 55) ↑ T2DM blood (53)^b^
Sphingosine 1-phosphate	HMDB0000277^^^	↑ T2DM blood (53)^b^
Steroids and steroid derivatives		
5a-Androstan-3a,17a-diol	HMDB0000458	↓ T2DM blood (53)^b^
Androsterone glucuronide	HMDB0002829	↓ T2DM blood (53)^b^
Androsterone sulfate	HMDB0002759	↓ T2DM blood (53)^b^
Chenodeoxycholic acid	HMDB0000518	↓ T2DM stool (49) ↑ T2DM blood (47)
Cholestane-3b-5a,6b-triol	HMDB0003990	↑ PreDM stool (41)
Cholesterol	HMDB0000067	↓ T2DM blood (2)
Cholic acid	HMDB0000619	↓ GDM blood (34), T2DM stool (46, 49) ↑ T2DM blood (47)
7-Dehydrodesmosterol	HMDB0003896	↑ PreDM stool (41), T2DM stool (41)
Dehydroepiandrosterone sulfate	HMDB0001032	↓ T2DM blood (53)^b^
Dehydrolithocholic acid	5283906	↓ GDM blood (34)
Deoxycholic acid	HMDB0000626	↑ T2DM blood (47)
Deoxycholic acid 3-glucuronide	HMDB0002596	↑ T1DM blood (29)
Dihydroxyvitamin D3	HMDB0000430	↓ T2DM blood (53)^b^
6,7-Diketolithocholic acid	137333800	↑ GDM blood (34)
Estrone glucuronide	HMDB0004483	↑ PreDM stool (41)
Glycochenodeoxycholic acid	HMDB0000637	↓ T2DM stool (49) ↑ PreDM blood (39)^d^
Glycocholic acid	HMDB0000138	↓ T2DM stool (46) ↑ T2DM blood (47)
Glycodeoxycholic acid	HMDB0000631	↓ T2DM stool (49) ↑ T2DM blood (47)
Glycolithocholic acid	HMDB0000698	↓ T2DM stool (49)
Glycoursodeoxycholic acid	HMDB0000708	↓ T2DM stool (46, 49) ↑ GDM blood (35)
Hyodeoxycholic acid	HMDB0000733	↑ GDM blood (34)
Isodeoxycholic acid	HMDB0002536	↑ GDM blood (34)
Lithocholic acid	HMDB0000761	↑ GDM blood (34), T1DM stool (27)
Physalolactone B	HMDB0034200	↓ T2DM stool (44)
Scillaren A	441870	↑ PreDM stool (41)
Taurochenodeoxycholic acid	HMDB0000951	↑ T2DM blood (47)
Taurodeoxycholic acid	HMDB0000896	↓ T2DM stool (49) ↑ T2DM blood (47)
Teasterone	13475125	↑ PreDM stool (41)
Taurohyodeoxycholic acid	119046	↑ GDM blood (34)
Tauroursodeoxycholic acid	HMDB0000874	↓ T2DM stool (49)
Taurolithocholate sulfate	HMDB0002580	↑ GDM blood (35)
Taurolithocholic acid	HMDB0000722	↓ T2DM stool (49)
Ursodeoxycholic acid	HMDB0000946	↓ T2DM stool (49)
Stilbenes		
Piceid	HMDB0030564	↑ T2DM stool (41)
Tetrapyrroles and derivatives		
Biliverdin	HMDB0001008	↓ T2DM blood (53)^b^

Arrows indicate direction of metabolite regulation in diabetes cohorts, relative to non-diabetes cohorts: increased (↑), decreased (↓), or reported as both increased or decreased depending on carbon structure or metabolite species (↕). Metabolites grouped by chemical class or subclass (according to HMDB) and recorded with sample type used in metabolomic analyses. Metabolites listed if reported as significantly discriminative between cohorts; with statistical significance recognised at p < 0.05, or threshold specified in-text based on independent correction analysis. **^a^** Independent components or carbon configuration not distinguished by retention time in all publications; **^b^** Correlation with DM is inferred based on significant associations with an alternate model predictive of diabetes, such as high HOMA-IR or glucose dispostition index; **^c^** Denotes findings from serum samples in instances of contradictory findings of metabolite levels in blood components; **^d^** Reported as trend between cohorts, no statistical significance reported in publication; **^^^** Example HMDB ID number provided, accession numbers vary depending on carbon structure or metabolite species. GDM, gestational diabetes mellitus; HMDB, Human Metabolome Database; PreDM, prediabetes mellitus; T1DM, type 1 diabetes mellitus; T2DM, type 2 diabetes mellitus; TUDCA, Tauroursodeoxycholic acid.

**Figure 3:**
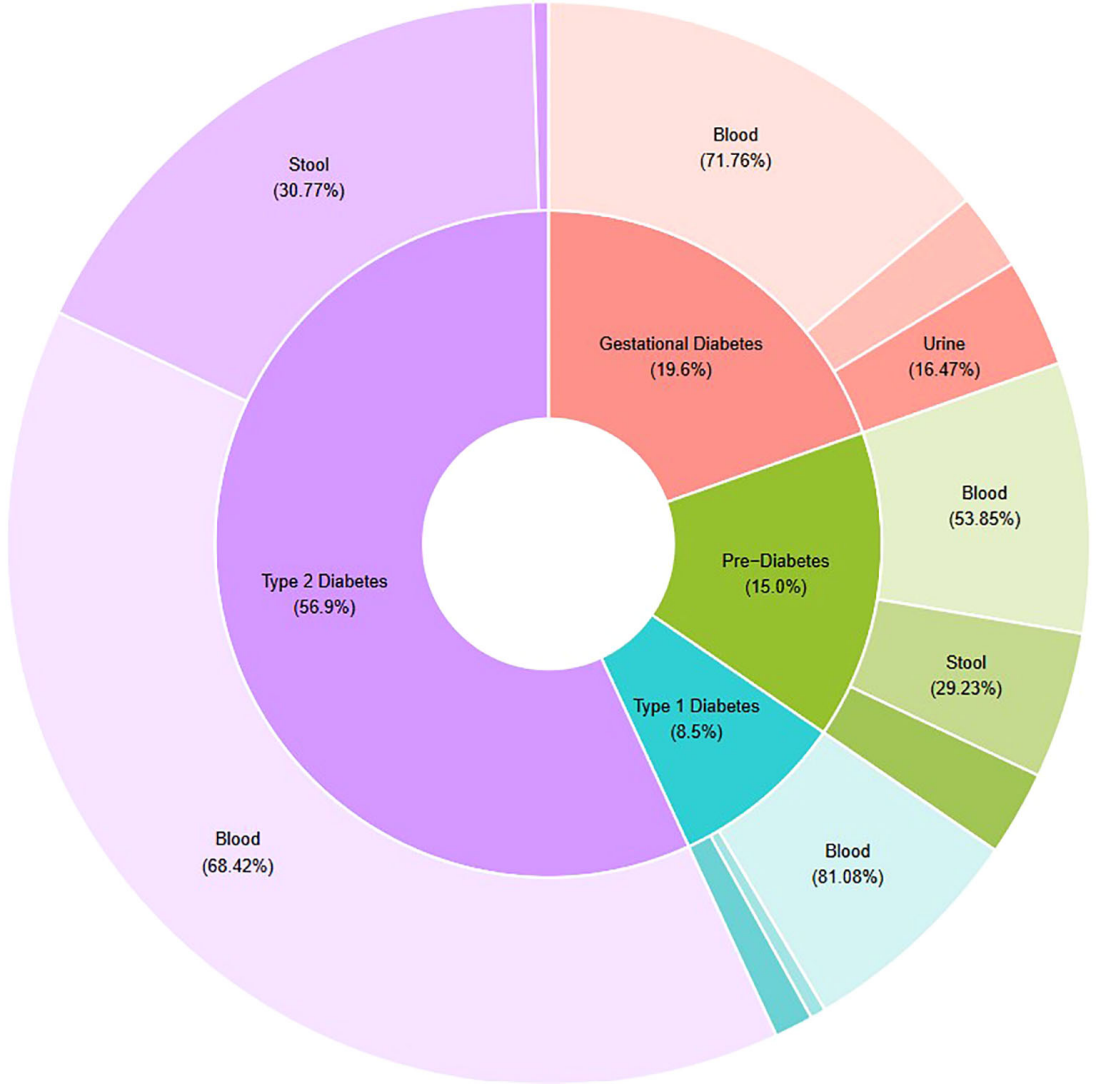
Schematic representing research targets of thirty-four studies analysed in this systematic review. Relative proportions based on counts of metabolites considered significantly different between cohorts depending on diabetes disease type (inner ring) and biological sample type (outer ring). Figure developed using R Studio software.

**Figure 3** demonstrates the proportions of reviewed studies that investigated each DM disease (inner ring), and, within each disease type, the proportion of studies that incorporated blood, urine and/or stool samples in metabolomic analyses (outer ring). The relative proportions depicted in **Figure 3** are based on the number of metabolites (targets listed in **Table 2**) each study reported as being significantly associated with DM.

### Summary of cohort descriptors

Thirty-four articles were analysed in this systematic review. The concentration of gut metabolites in individuals with T1DM was investigated in four studies (26–29); seven studies investigated GDM cohorts (30–36); eight studies investigated PreDM, or PreDM and T2DM cohorts (2, 37–43); and fifteen studies investigated T2DM cohorts only (44–58). Given the pre-determined exclusion criteria for this systematic review, all thirty-four studies incorporated an observational experimental design, with no interventional input. Cohort sizes varied significantly between studies depending on study objectives and stringency of inclusion/exclusion criteria. As depicted in **Figure 3**, across all diabetes diseases, the majority of metabolomic findings were obtained from blood components (plasma and/or serum). However, as outlined in **Table 1**, eleven studies analysed two types of biological samples (plasma and urine, plasma and stool, plasma and serum, stool and urine, or stool and serum).

### Summary of analysis descriptors

Of the thirty-four studies reviewed, fifteen employed targeted metabolomic approaches; the majority of which targeted bile acids (BA), short-chain fatty acids (SCFA), or trimethylamine N-oxide (TMAO) and precursor metabolites. Fourteen articles incorporated untargeted metabolomic analyses, while an additional five studies combined targeted and untargeted metabolomic approaches. The details of each metabolomic method are outlined in **Table 1**. Briefly, mass spectrometry (MS) analyses were the most widely employed method; specifically, liquid chromatography-mass spectrometry configurations were employed in thirty-seven analyses (75.5%), gas chromatography-mass spectrometry methods were utilised in nine articles (18.3%), while capillary electrophoresis-mass spectrometry was used in a single study (2.5%). Nuclear magnetic resonance (NMR) metabolomic analyses were incorporated in five analyses (12.8%). Differential mobility spectroscopy and high-performance liquid chromatography with ultra-violet spectroscopy were each utilised in one analysis (2.5% per method).

### Summary of metabolomic findings

Analysis of metabolomic findings revealed a total of 272 metabolites, across thirty-eight chemical classes and sub-classes, determined to be significantly associated with incidence of diabetes. Amino acid compounds were the most widely identified across all diabetes diseases; whilst only single metabolite targets were identified from several smaller classes of organoheterocyclic compounds (such as lactones and dihydrofuran compounds). The metabolites most widely cited as elevated in the reviewed studies were amino acids, leucine, and isoleucine. In contrast, levels of the sphingolipid metabolite, sphingomyelin, were most consistently observed to be decreased, with three articles reporting down-regulation in T2DM cohorts, relative to non-diabetes controls. As **Table 2** depicts, consistent reporting of increased or decreased metabolite levels was predominantly observed in T2DM cohorts only; across other diabetes diseases, reported metabolites were only supported by single findings. This also reflects the trend depicted in **Figure 3**, in which the largest metabolite profiles were obtained from studies of T2DM (59.22%).

**Figure 4:**
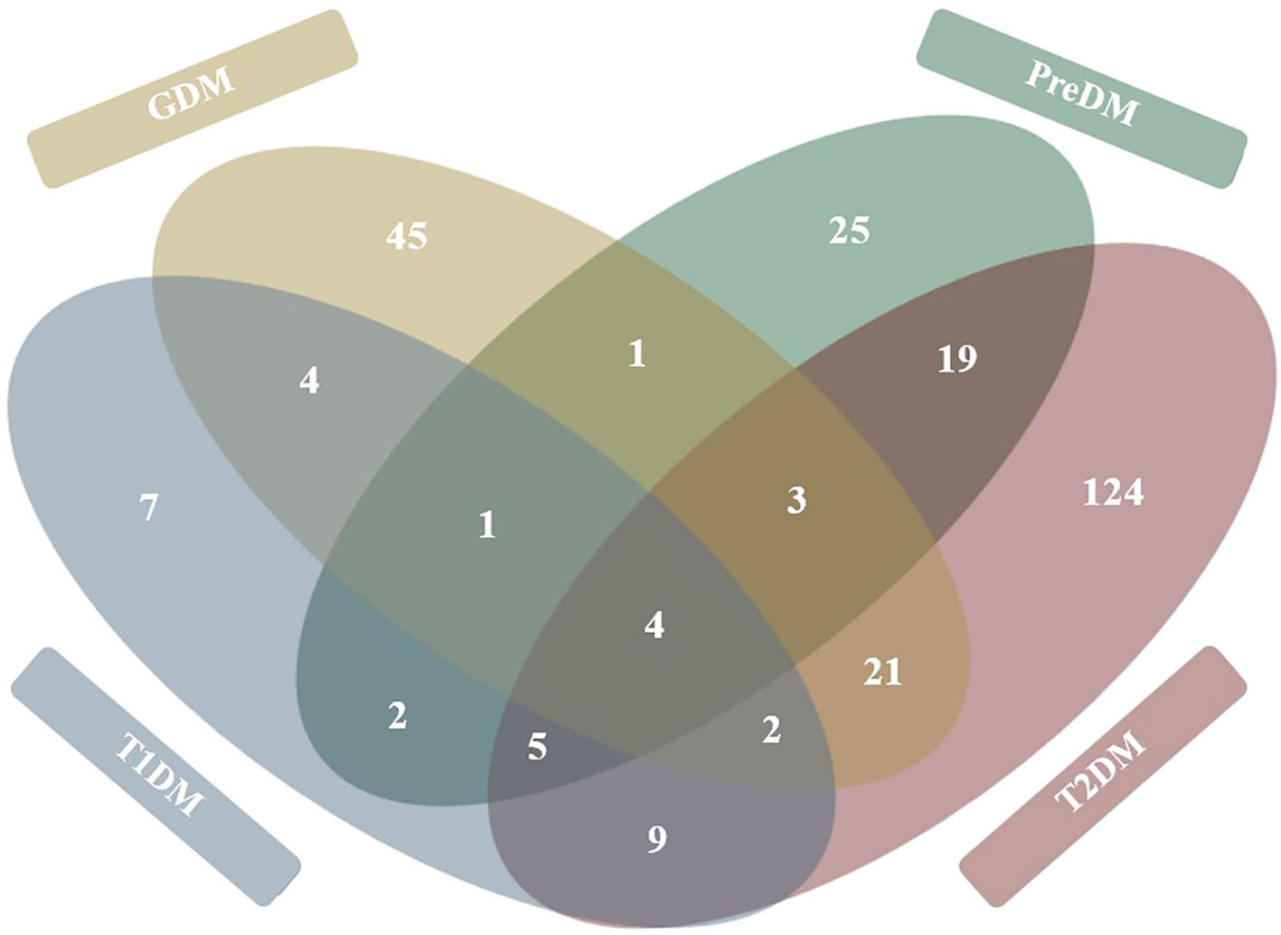
Venn diagram representation of metabolites significantly associated with diabetes mellitus diseases. Figure represents 272 total metabolites reported as being significantly associated (either positively or negatively) with each of the four diabetes disease types. Metabolites shared between diabetes diseases are tallied in overlapping circles. Figure developed based on findings of thirty-four primary research articles reviewed in systematic review.

**Figure 5:**
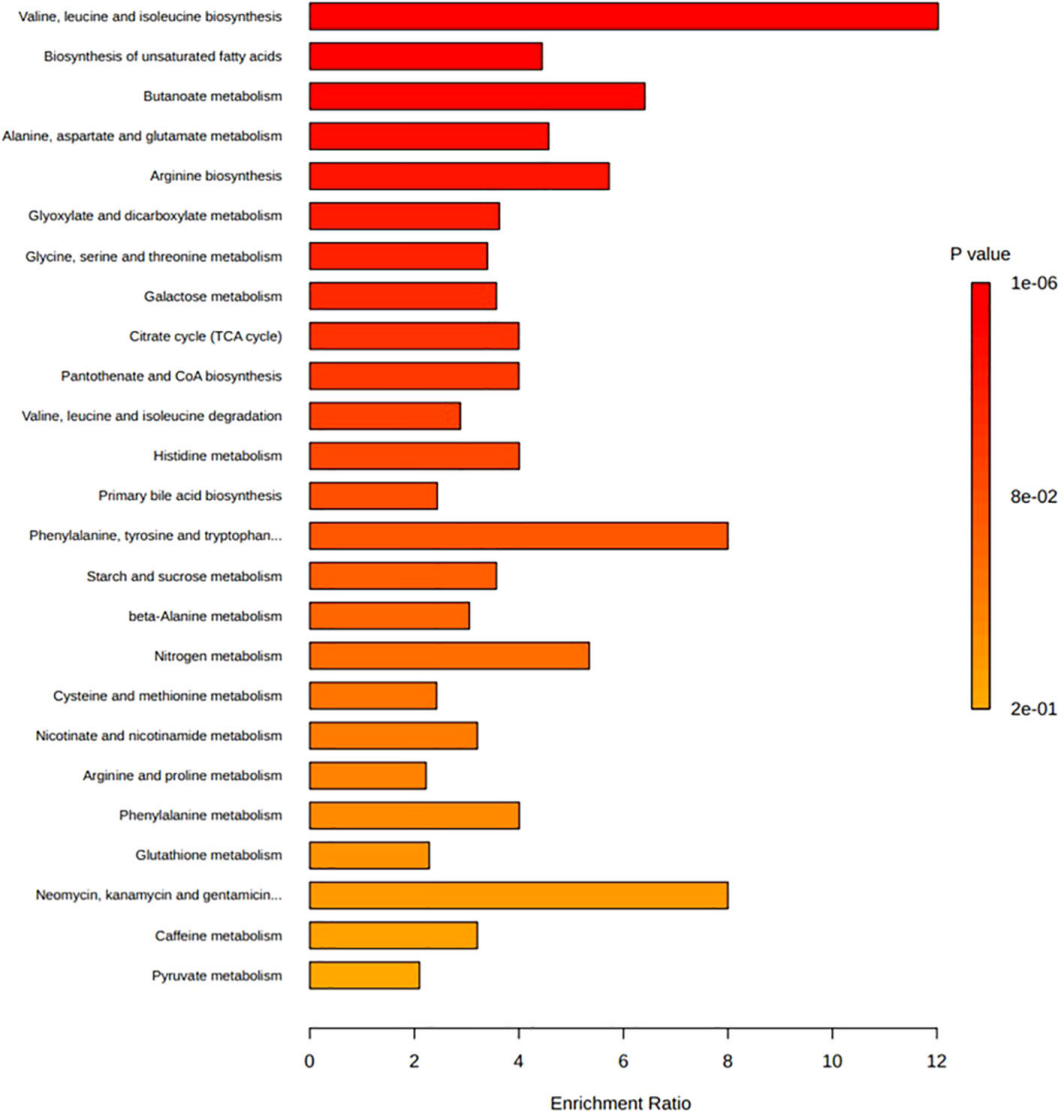
Enrichment ratio and p-value from enrichment analyses of the top twenty-five pathways associated with those metabolites reported as being significantly associated with diabetes diseases. Bar lengths represent enrichment ratio, calculated by the number of hits divided by the expected number of hits within each pathway. Enrichment analysis conducted on metabolites determined to be significantly associated with incidence of diabetes disease (**Table 2**) and those with primary accession numbers indexed in the HMDB library. Enriched metabolic pathways are ordered according to decreasing p-value. Metabolite pathways defined based on KEGG human metabolic pathways library. Enrichment analysis conducted and visualised by MetaboAnalyst program.

Analysis of the core metabolome shared between all the diseases revealed four metabolites to be significantly associated with T1DM, GDM, PreDM and T2DM cohorts (**Figure 4**). Of these, the alpha-hydroxy acid, 2-hydroxybutyric acid and the branched-chain amino acid (BCAA), valine, were determined to be consistently elevated. Whilst the amino acid, alanine, and BCAAs, leucine/isoleucine were also reported across all disease types, discrepancies existed between studies as to whether these compounds were increased or decreased in T1DM cohorts. In addition to these core metabolites, a further eleven targets were identified as being shared across three of the diabetes diseases (**Figure 4**). These included glucose, mannose, 3-hydroxybutyric acid, palmitic acid, and ketoisocaproic acid which were all reported as being significantly elevated across three different diabetes disease types. Tyrosine, tryptophan, palmitoleic acid, phosphatidylcholines, phosphatidylcholines, and triglycerides were also reported across three diabetes diseases, but findings of increased or decreased levels were discrepant between articles, depending on the sample type used in downstream metabolomic analyses. Pathways involved in the biosynthesis of BCAAs valine, leucine and isoleucine were determined to have the highest enrichment ratio and strongest enrichment p-value; followed by pathways involved in the biosynthesis of phenylalanine, tyrosine and tryptophan, and unsaturated fatty acids (**Figure 5**). Biosynthesis of the alpha-amino acid, tryptophan, is a highly enriched metabolic pathway (**Figure 5**), and the metabolite is also shown to share a high number of metabolic relationships within the network (degree = 96, betweenness = 10279.40).

**Figure 5** depicts the enrichment ratios and p-values from enrichment analyses conducted on the metabolites reported as being significantly associated with the incidence of T1DM, GDM, PreDM and T2DM (**Table 2**). Bar plot depicts the top twenty-five metabolite pathways (according to calculated p-value) enriched in diabetes disease, based on the gut-associated metabolites collated in this systematic review. Data and corresponding metabolite-metabolite network for this enrichment analysis are presented in **Supplementary Table S1**.

Across all four diabetes diseases, discrepancies were reported between articles as to the concentrations of metabolites in the same, and different sample types. For example, fatty acids butyrate, heptadecenoic acid, and myristic acid were reported as both increased and decreased in blood samples collected from T2DM cohorts (relative to respective non-diabetes cohorts); but were also reported to be elevated in stool samples from T2DM cohorts. Further exemplifying this discrepancy; the concentration of key bile acids glycodeoxycholic acid, glycocholic acid, cholic acid and chenodeoxycholic acid were reported to be elevated in blood samples but decreased in stool samples collected from T2DM cohorts. The concentrations of four metabolites was dependent on carbon configuration, isomer structure or metabolite species: namely, ceramide, tridecanoic acid, and phosphatidylethanolamine and triglyceride compounds.

### Specimen type used in studies of diabetes correlates with disease type and metabolite regulation findings

Chi-Square tests of association revealed evidence of a significant correlation between the type of specimen used in metabolomic analysis, and whether metabolite levels were reported as being increased or decreased (p = 3.5e-8). Incorporation of stool samples was significantly associated with identification of decreased metabolite levels; whilst the opposite was true of studies that utilised blood samples, as this specimen type was significantly associated with the identification of increased metabolite levels.

Further Chi-Square testing revealed a significant correlation between the type of diabetes and specimen used in metabolomic analyses (p = 1.4e-9). *Post-hoc* testing of this correlation revealed significant associations between two groups; GDM studies identified metabolites more from urine biospecimens, while T2DM studies identified metabolites more from stool and less from urine samples. Finally, statistical analyses revealed no significant association between type of diabetes disease and whether metabolite levels were reported as being increased or decreased.

## Discussion

Metabolic disorders, particularly T1DM, GDM and T2DM, are physiologically distinct diseases, with very different causes, treatments, and outcomes. As such, the aim of this review was not to directly compare these aetiologies, but to collectively synthesise published metabolomics data and identify common disease targets; thereby maximising the future research potential of identified biomarkers. In achieving this, our review identified a core metabolomic signature that is common to all DM diseases. The four metabolites that comprise this shared diabetes metabolome are the alpha hydroxy acid, 2-hydoxybutyric acid, the amino acid, alanine, and BCAAs, valine, and the leucine/isoleucine isomer system (**Table 2**, **Figure 4**). As a by-product of protein metabolism, 2-hydroxybutyric acid has been positively correlated to the impairment of pancreatic β-cells (59) as a well-established early hallmark of insulin sensitivity, and resistance (60). Corroborating this, the findings of our review showed the metabolite to be elevated in blood samples taken from T1DM (26), GDM (61, 62), PreDM (2), and T2DM (48) cohorts. Similarly, concentrations of valine were significantly higher across all DM diseases in blood (2, 27, 40, 42, 55), stool (63), and urine (29); an expected finding given valine is widely associated with the incidence of oxidative stress, decreased insulin secretion, and high glucose levels (64). In contrast to 2-hydroxybutyric acid and valine, the role of leucine and isoleucine in DM is controversial. Some studies report that, alongside valine, the BCAAs have a detrimental effect on human metabolic health by decreasing insulin secretion; and even linking isoleucine with increased T2DM risk (65). However, other studies report that the metabolite acutely stimulates insulin production and plays an important role in ameliorating adiposity and maintaining glucose homeostasis (66, 67). In our review, the concentration of leucine and isoleucine were found to be elevated in the blood (2, 27, 34, 40, 42, 51, 55, 58) and urine (29) of individuals with DM, whilst leucine was reported to be decreased in T1DM cohorts (26). These contradictory findings reiterate the need for future studies that provide clarity around the regulatory profiles of these BCAAs in different biological sample types, and across the spectrum of diabetes diseases.

The majority of studies analysed in this review characterised the human intestinal metabolome associated with T2DM (**Figures 3**, **4**). The focus of research on T2DM is commensurate with its high prevalence (86.4% of Australasian DM diagnoses (2, 27, 34, 40, 42, 51, 55, 58)). Our review has drawn attention to the unmet need for GDM- and T1DM-specific metabolomic research, given the distinct pathophysiological processes associated with the development of DM diseases. Our review has also highlighted the importance of appropriate interpretation of GDM studies; ensuring any significant findings are drawn from comparisons of appropriate disease and control cohorts. This is best exemplified in the findings of Ivanovova et al. (2021), who demonstrated significant differences in the levels of multiple SCFAs between GDM and non-pregnant cohorts, but not between GDM and pregnant-non-GDM cohorts. For future studies in the field of GDM-research, this highlights the importance of incorporating proper phenotype controls in study designs. Doing so will ensure conclusions can be drawn regarding the impact of GDM, and not pregnancy itself; a period that is already well-known to involve significant changes to the female gut microbiota and metabolome (33).

Our findings reinforce that biological sample type has a significant impact on metabolomic findings. This is best exemplified by comparing independent findings published by Zhou et al. (2019, 2020) (68, 69); which indicated that the levels of the same BA and SCFA metabolite targets were elevated in serum yet decreased in stool collected from the same participant cohorts. Overall, our review identified twenty-seven metabolites that were both increased and decreased, depending on whether blood, urine or stool was used in metabolomic analyses (**Table 2**). BAs were the most frequently discrepantly reported chemical species; with decreased levels in stool samples, yet increased levels in blood samples from DM cohorts. A site-specific difference is expected given only an estimated 5% of primary and secondary BAs are excreted in stool, while up to 95% are reabsorbed by the terminal ileum and transported back to the liver via portal circulation (62). However, literature reports that diabetes initiates changes in BA metabolism and composition (70, 71), and is accompanied by an increase in BA stool excretion; which does not support the decreased levels seen in stool profiles reported in the reviewed studies. Such discrepancies highlight the potential bias that sample type and collection methods may impose on metabolomic findings and emphasise that researchers should consider the inherent advantages and disadvantages of each sample type when designing studies that target human- and microbial-derived metabolites in the gut.

All results incorporated in this systematic review were based on findings from non-invasive, or minimally invasive sample types including stool, urine, and blood components (plasma/serum). Across all four DM diseases, blood was the most utilised sample, with stool the second most frequently utilised in T2DM and PreDM studies, and urine the second most utilised in T1DM and GDM studies (**Figure 3**). The incorporation of urine sample collection from T1DM and GDM cohorts is pragmatic in the clinical context given the often routine requirement for these patient groups to submit samples for urine ketone, protein and albumin testing, and hence easier recruitment to research studies. However, the discrepancies reported here, and in studies such as that of Deng, Xu, Shen, et al. (2023) (48), underline the importance of careful selection of sample types to ensure results provide the most accurate representation of the metabolome at the target organ site. Researchers may also consider the use of other sample types to analyse host-gut interactions, such as saliva, exhaled breath, or invasive sample types, such as tissue biopsies (26).

This systematic review also reported discrepant findings in the levels of twenty-six metabolites between studies that incorporated the same sample type in downstream metabolomic analyses (**Table 2**). These findings highlight the need for guidelines that standardise collection methods for biological samples used in metabolomic analyses. This could include best practice recommendations for sample collection times (such as first/second morning urine sampling), the preferred use of single-batch consumables to prevent inter-batch variability generating inconsistent artefacts, and recommendations to reduce contamination in tissue collection (for example, using protected specimen brush techniques in mucosal-luminal sampling) (68, 69). Enforcing the standardisation of these collection and preparation methods will work to limit inter-sample variability and maintain the biological and metabolic integrity of samples consistently across study cohorts (68).

In addition to sample type selection, pre-analytical procedures are well known to influence microbiome and metabolome readouts. In particular, collection and storage methods have a well-established impact on the accuracy and precision of downstream metabolomic results (65, 72), and metabolite extraction technique/s are critical to either preventing or causing biases in results (73). The majority of studies included in this review reported sample handling procedures in line with best practice guidelines (74) (**Table 1**). However, given the different sample types, chemical targets, metabolomic approaches and MS or NMR instrumentation used in each of the thirty-four reviewed studies (**Table 1**), the pre-treatment extraction and derivisation processes varied widely, and may explain discrepancies between studies. Therefore, while sample preparation methods have been comprehensively reviewed in the literature (29), further comparative analyses are required to elucidate inter-method and inter-platform biases.

Much of the research reviewed in this study reported differential concentrations of targeted metabolites; however, it is not made clear whether these markers were differentially increased or decreased between cohorts. Additional discrepancies also exist in the reporting of metabolite marker regulation; further confusing the role these metabolites play as either a ‘cause or cure’ to diabetes. This iterates the need for standardised reporting in future clinical metabolomics publications. Potential measures for implementation may include standardised reporting of fold changes between cohorts, as well as cross-referencing and reporting targets alongside primary accession codes linked to a publicly accessible database, such as HMDB, KEGG, PubChem, ChEBI, or UniProt. Doing so will prevent chemically synonymous compounds being misreported as novel targets and maximise the research potential of future studies in the metabolomics field.

A limitation of this review is the comparison of metabolite concentrations on a dichotomous scale; increased or decreased concentrations depending on healthy or diabetes status. Performing a quantitative comparison of metabolomic concentrations across the various study cohorts falls outside the scope of this systematic review and warrants further study. Therefore, further meta-analysis of the metabolomics data synthesised here is required to quantitatively compare metabolite concentrations; taking into consideration the inter-method effects of metabolomics tools, platform-specific biases, and the array of analytical pipelines consulted by researchers. Future statistical analysis should also consider controlling for participant baseline characteristics that vary from study to study; for instance, age, sex, duration of diabetes, race, genetic background, diet/lifestyle factors, or the use of any antidiabetic treatments or known microbiome-altering medications and supplements. Furthermore, the results of this review do not account for metabolome changes over time, or those stemming from diabetes-associated comorbidities (such as diabetic nephropathy, neuropathy, or retinopathy) which are known to further perturb the gut microbiota and metabolism (44, 45). It would therefore be pertinent for future studies to consider the effects attributable to the incidence and stage of comorbidities, by sub-stratifying participant cohorts accordingly. The results of our review may also be considered in conjunction with future microbiome-metabolome correlative analyses that incorporate tools such as 16S ribosomal RNA gene sequencing or whole genome shotgun sequencing. Alternatively, publicly available datasets, such as that curated by Muller, Algavi, and Borenstein (2022) (75) from faecal samples, provide fully processed, and benchmarked microbiome-metabolome integration tools to enable correlative analyses. Linking metabolomic outputs to microbial profiles is a key future research target, as it will provide a deeper insight into the functional capacity of the microbiota.

## Conclusions

In conclusion, this systematic review analysed the findings of thirty-four articles investigating the intestinal metabolome associated with the incidence of T1DM, GDM, PreDM, and T2DM. Extracted data mapped the concentrations of 272 intestinal metabolites across thirty-eight chemical classes and sub-classes. To date, the majority of diabetes metabolome research has been conducted in T2DM cohorts; and while the experimental, and clinical importance of these studies is undeniable, this review highlights a bias in the research foci that has left GDM and T1DM diseases understudied. Our review also described a novel comparison of metabolite levels between blood, stool, and urine samples in DM cohorts. This is significant given few studies have investigated the potential biasing effect of sample type on the downstream identification of primary and secondary metabolites. The results of this work, and of other newly emerging research in the field (64), urge caution in directly inferring associations between the stool metabolome and the incidence of systemic diseases, such as diabetes. Overall, the key metabolites identified in this review warrant further investigation as potential diagnostic biomarkers or targets in the treatment of DM.

## Data Availability

The original contributions presented in the study are included in the article/**Supplementary Material**. Further inquiries can be directed to the corresponding author.
