# Supplementary material for: Gut-associated metabolites and diabetes pathology: a systematic review
Source: Front Endocrinol (Lausanne). 2025 May 21;16:1559638. doi: 10.3389/fendo.2025.1559638 (PMC12133462; doi:10.3389/fendo.2025.1559638)
Supplement: Supplementary file 1 [file Table1.docx]

Supplementary Material

**Supplementary Table 1. Overview of enrichment analyses conducted on metabolites reported as significantly associated with diabetes diseases.** Heatmapping corresponds to metabolite enrichment ratio bar plots in Figure 11. Analyses conducted using MetaboAnalyst program.

| **Heatmap corresponding to enrichment ratio** | **Metabolite Set** | **Total hits** | **Expected hits** | **P value** | **Holm P** | **FDR** | **Details** |
| --- | --- | --- | --- | --- | --- | --- | --- |
| ﻿ | Valine, leucine and isoleucine biosynthesis | 8 | 6 | 0.499 | 1.28E-6 | 1.02E-4 | 1.02E-4 |
| ﻿ | Biosynthesis of unsaturated fatty acids | 36 | 10 | 2.25 | 3.66E-5 | 0.0029 | 0.00147 |
| ﻿ | Butanoate metabolism | 15 | 6 | 0.936 | 1.6E-4 | 0.0125 | 0.00377 |
| ﻿ | Alanine, aspartate and glutamate metabolism | 28 | 8 | 1.75 | 1.89E-4 | 0.0145 | 0.00377 |
| ﻿ | Arginine biosynthesis | 14 | 5 | 0.873 | 0.00109 | 0.0826 | 0.0174 |
| ﻿ | Glyoxylate and dicarboxylate metabolism | 31 | 7 | 1.93 | 0.00225 | 0.169 | 0.03 |
| ﻿ | Glycine, serine and threonine metabolism | 33 | 7 | 2.06 | 0.0033 | 0.244 | 0.0377 |
| ﻿ | Galactose metabolism | 27 | 6 | 1.68 | 0.00511 | 0.373 | 0.0499 |
| ﻿ | Citrate cycle (TCA cycle) | 20 | 5 | 1.25 | 0.00623 | 0.449 | 0.0499 |
| ﻿ | Pantothenate and CoA biosynthesis | 20 | 5 | 1.25 | 0.00623 | 0.449 | 0.0499 |
| ﻿ | Valine, leucine and isoleucine degradation | 39 | 7 | 2.43 | 0.00872 | 0.61 | 0.0634 |
| ﻿ | Histidine metabolism | 16 | 4 | 0.998 | 0.0145 | 0.999 | 0.0965 |
| ﻿ | Primary bile acid biosynthesis | 46 | 7 | 2.87 | 0.0212 | 1.0 | 0.118 |
| ﻿ | Phenylalanine, tyrosine and tryptophan biosynthesis | 4 | 2 | 0.25 | 0.0213 | 1.0 | 0.118 |
| ﻿ | Starch and sucrose metabolism | 18 | 4 | 1.12 | 0.0221 | 1.0 | 0.118 |
| ﻿ | beta-Alanine metabolism | 21 | 4 | 1.31 | 0.0375 | 1.0 | 0.187 |
| ﻿ | Nitrogen metabolism | 6 | 2 | 0.374 | 0.049 | 1.0 | 0.223 |
| ﻿ | Cysteine and methionine metabolism | 33 | 5 | 2.06 | 0.0502 | 1.0 | 0.223 |
| ﻿ | Nicotinate and nicotinamide metabolism | 15 | 3 | 0.936 | 0.0619 | 1.0 | 0.261 |
| ﻿ | Arginine and proline metabolism | 36 | 5 | 2.25 | 0.0688 | 1.0 | 0.275 |
| ﻿ | Phenylalanine metabolism | 8 | 2 | 0.499 | 0.0843 | 1.0 | 0.321 |
| ﻿ | Glutathione metabolism | 28 | 4 | 1.75 | 0.0921 | 1.0 | 0.335 |
| ﻿ | Neomycin, kanamycin and gentamicin biosynthesis | 2 | 1 | 0.125 | 0.121 | 1.0 | 0.417 |
| ﻿ | Caffeine metabolism | 10 | 2 | 0.624 | 0.125 | 1.0 | 0.417 |
| ﻿ | Pyruvate metabolism | 23 | 3 | 1.43 | 0.169 | 1.0 | 0.541 |
| ﻿ | Glycerophospholipid metabolism | 36 | 4 | 2.25 | 0.183 | 1.0 | 0.562 |
| ﻿ | Pyrimidine metabolism | 39 | 4 | 2.43 | 0.222 | 1.0 | 0.659 |
| ﻿ | Lipoic acid metabolism | 28 | 3 | 1.75 | 0.252 | 1.0 | 0.72 |
| ﻿ | Linoleic acid metabolism | 5 | 1 | 0.312 | 0.276 | 1.0 | 0.76 |
| ﻿ | Porphyrin metabolism | 31 | 3 | 1.93 | 0.304 | 1.0 | 0.812 |
| ﻿ | Pentose and glucuronate interconversions | 19 | 2 | 1.19 | 0.334 | 1.0 | 0.841 |
| ﻿ | Fatty acid biosynthesis | 47 | 4 | 2.93 | 0.336 | 1.0 | 0.841 |
| ﻿ | Fructose and mannose metabolism | 20 | 2 | 1.25 | 0.358 | 1.0 | 0.855 |
| ﻿ | Thiamine metabolism | 7 | 1 | 0.437 | 0.363 | 1.0 | 0.855 |
| ﻿ | Propanoate metabolism | 21 | 2 | 1.31 | 0.381 | 1.0 | 0.87 |
| ﻿ | Taurine and hypotaurine metabolism | 8 | 1 | 0.499 | 0.403 | 1.0 | 0.896 |
| ﻿ | Ascorbate and aldarate metabolism | 9 | 1 | 0.561 | 0.441 | 1.0 | 0.918 |
| ﻿ | Purine metabolism | 70 | 5 | 4.37 | 0.446 | 1.0 | 0.918 |
| ﻿ | Biotin metabolism | 10 | 1 | 0.624 | 0.476 | 1.0 | 0.918 |
| ﻿ | Tryptophan metabolism | 41 | 3 | 2.56 | 0.477 | 1.0 | 0.918 |
| ﻿ | Glycolysis / Gluconeogenesis | 26 | 2 | 1.62 | 0.49 | 1.0 | 0.918 |
| ﻿ | Tyrosine metabolism | 42 | 3 | 2.62 | 0.494 | 1.0 | 0.918 |
| ﻿ | Amino sugar and nucleotide sugar metabolism | 42 | 3 | 2.62 | 0.494 | 1.0 | 0.918 |
| ﻿ | Lysine degradation | 30 | 2 | 1.87 | 0.569 | 1.0 | 1.0 |
| ﻿ | alpha-Linolenic acid metabolism | 13 | 1 | 0.811 | 0.569 | 1.0 | 1.0 |
| ﻿ | Sphingolipid metabolism | 32 | 2 | 2.0 | 0.604 | 1.0 | 1.0 |
| ﻿ | Glycosylphosphatidylinositol (GPI)-anchor biosynthesis | 15 | 1 | 0.936 | 0.621 | 1.0 | 1.0 |
| ﻿ | Glycerolipid metabolism | 16 | 1 | 0.998 | 0.645 | 1.0 | 1.0 |
| ﻿ | Ubiquinone and other terpenoid-quinone biosynthesis | 18 | 1 | 1.12 | 0.688 | 1.0 | 1.0 |
| ﻿ | Fatty acid degradation | 39 | 2 | 2.43 | 0.712 | 1.0 | 1.0 |
| ﻿ | Ether lipid metabolism | 20 | 1 | 1.25 | 0.727 | 1.0 | 1.0 |
| ﻿ | Steroid biosynthesis | 41 | 2 | 2.56 | 0.738 | 1.0 | 1.0 |
| ﻿ | Steroid hormone biosynthesis | 87 | 4 | 5.43 | 0.807 | 1.0 | 1.0 |
| ﻿ | Inositol phosphate metabolism | 30 | 1 | 1.87 | 0.858 | 1.0 | 1.0 |
| ﻿ | Fatty acid elongation | 38 | 1 | 2.37 | 0.916 | 1.0 | 1.0 |
| ﻿ | Arachidonic acid metabolism | 44 | 1 | 2.74 | 0.944 | 1.0 | 1.0 |
| ﻿ | Drug metabolism - cytochrome P450 | 55 | 1 | 3.43 | 0.973 | 1.0 | 1.0 |
